# Associations Between the Chippaux–Smirak Index and Clinical Foot Assessments in Young Adults: A Cross-Sectional Study

**DOI:** 10.3390/diagnostics16213455

**Published:** 2026-10-22

**Authors:** Amneh Alshawabka, Mahmoud Alfatafta, Huthaifa Atallah, Fahad Alhazmi, Mohannad Alkhateeb, Anthony McGarry, Wa’el Qa’dan

**Affiliations:** 1 Department of Prosthetics and Orthotics, School of Rehabilitation Sciences, The University of Jordan, Amman 11942, Jordan; 2 Department of Health Service and Hospital Administration, Faculty of Economics and Administration, King Abdulaziz University, Jeddah 21589, Saudi Arabia; 3 School of Public Health and Community Medicine, Institute of Medicine, University of Gothenburg, 40530 Gothenburg, Sweden; 4 Biomedical Engineering Department, University of Strathclyde, Glasgow G4 0NW, UK; 5 3D Printing and Visualisation Centre, Medical School, University of Pécs, 7624 Pécs, Hungary

**Keywords:** Chippaux–Smirak Index, foot posture index, medial longitudinal arch, navicular drop, resting calcaneal stance position, hallux valgus, lesser toe deformities

## Abstract

**Background/Objectives**: The Chippaux–Smirak Index (CSI) measures relative midfoot plantar contact, whereas the six-item Foot Posture Index (FPI-6), navicular drop test (NDT), and resting calcaneal stance position (RCSP) assess static foot posture, change in navicular height, and calcaneal alignment, respectively. This study examined associations among these measures, agreement between CSI and FPI-6 classifications, and measurement reliability in young adults. **Methods**: This cross-sectional study included 350 young adults, with both feet assessed (700 feet). Two orthotists independently measured CSI, FPI-6, NDT, and RCSP. Generalised estimating equation models accounted for correlations between feet and were adjusted for age, sex, body mass index, and foot side. Agreement was assessed using exact percentage agreement and linear weighted Cohen’s kappa, and reliability using intraclass correlation coefficients (ICCs). **Results**: After adjustment, each increase of 10 percentage points in CSI was associated with a 2.141-point-higher FPI-6 total score, a 2.660 mm greater navicular drop, and an RCSP that was 3.240° more valgus (all *p* < 0.001). CSI and grouped FPI-6 classifications agreed for 82.9% of feet (linear weighted κ = 0.664, 95% CI 0.595–0.727). Single-measure interrater ICCs ranged from 0.644 for NDT to 0.884 for CSI. Digital remeasurement of CSI demonstrated excellent intrarater reliability (ICC(A,1) = 0.989). **Conclusions**: Higher CSI values were associated with a more pronated static foot posture, greater navicular drop, and a more valgus RCSP. Incomplete agreement between CSI and FPI-6 classifications indicates that these methods provide related but distinct information and should be interpreted according to the specific foot characteristic assessed.

## 1. Introduction

The medial longitudinal arch contributes to load distribution within the foot and shock absorption [1,2]. Characterising arch morphology is relevant in clinical assessment and research because variation in arch structure and plantar contact has been associated with differences in plantar loading, foot posture and foot function [1,2]. Foot posture and function have also been associated with several foot disorders [3].

Arch morphology is commonly described as a high, normal or low arch [1,2]. A foot with a high arch is generally characterised by greater medial longitudinal arch height and reduced midfoot plantar contact [1,2]. In contrast, a foot with a low arch is generally characterised by reduced medial longitudinal arch height and greater midfoot plantar contact [1,2]. Several methods are available to assess the medial longitudinal arch, including assessments of clinical foot posture, plantar contact, foot alignment and changes in arch height during weight bearing [1,2,4]. Because these methods assess different characteristics of the foot, they may produce different classifications for the same foot [1,2,5]. A systematic review also identified variation in measurement methods and classification criteria, which limited conclusions about the validity and reliability of nonradiographic foot assessments [4].

Footprint analysis is a nonradiographic method used to assess plantar contact and characterise medial longitudinal arch morphology. Common footprint methods include Clarke’s angle, the Arch Index, the Staheli Index, the Harris Imprint Index and the Chippaux–Smirak Index (CSI) [1,2,6]. These methods quantify different features of the footprint: some measure an angle, some calculate the proportion of the plantar surface in contact with the ground, and others compare the widths of different footprint regions [1,2,6].

Foot classification may vary according to the footprint method and classification criteria used [1,2]. CSI, the Harris Imprint Index and the Staheli Index were also evaluated using the radiographic talar–first metatarsal angle as the reference measure [6]. The talar–first metatarsal angle is measured between the longitudinal axes of the talus and first metatarsal bone on a lateral radiograph obtained during weight bearing. CSI showed the highest accuracy for identifying a low arch based on the radiographic classification, but all three footprint methods demonstrated fair accuracy [6]. In the same study, intrarater and interrater agreement for footprint classifications ranged from moderate to almost perfect, with the highest agreement reported for CSI [6]. Another study of adults also reported high CSI reliability [7]. These findings support the use of CSI as a reproducible quantitative measure of relative midfoot plantar contact, although reproducibility does not necessarily indicate diagnostic accuracy [6,7]. CSI represents the narrowest midfoot contact width relative to the widest forefoot contact width [2,6,7]. Higher CSI values therefore represent greater relative midfoot contact width and are generally associated with a low arch footprint pattern, whereas lower values are associated with a high arch footprint pattern [6]. However, CSI reflects plantar contact rather than directly measuring skeletal arch alignment [1,2], and the resulting measurements may be influenced by plantar soft tissues [2,7].

Other assessment methods evaluate foot posture, arch height change and rearfoot alignment. The six-item Foot Posture Index (FPI-6) evaluates static foot posture, defined as the alignment of the foot segments during standing, through observations of the rearfoot, midfoot and forefoot [8,9]. It classifies foot posture as highly supinated, supinated, neutral, pronated or highly pronated. A supinated posture generally presents with a higher arch and an inverted rearfoot, whereas a pronated posture presents with a lower arch and an everted rearfoot [8,9]. Arch height change can be assessed using the navicular drop test (NDT), which measures the difference in navicular height between subtalar neutral and relaxed standing positions [10]. Rearfoot alignment can be assessed using resting calcaneal stance position (RCSP), which measures the frontal plane angle of the calcaneus relative to the vertical during relaxed standing [11]. Together, these measures provide information about relative midfoot plantar contact, overall static foot posture, arch height change and calcaneal alignment.

Previous studies have reported variable relationships among these assessment methods [5,7,12,13]. In adults, foot classifications based on FPI-6, NDT, rearfoot angle and medial longitudinal arch angle, measured at the navicular tuberosity between lines extending to the medial malleolus and first metatarsal head, showed moderate overall agreement [5]. A positive correlation has also been reported between NDT and CSI [7]. Among young adults with a low arch, CSI showed a good positive correlation with FPI-6, while a stronger positive correlation was observed between FPI-6 and NDT [12]. In children aged 5–8 years, FPI-6 was positively associated with CSI and NDT, with CSI showing the strongest association among the footprint methods evaluated [13].

Despite these findings, evidence examining the relationships among CSI, FPI-6, NDT and RCSP within the same sample of young adults remains limited. Musculoskeletal structure and biomechanics change during growth and maturation [14]. Foot dimensions and normalised arch height vary with age during childhood and adolescence [15]. Consistent with these developmental changes, longitudinal assessment using CSI showed a significant reduction in relative midfoot plantar contact width between four and five years of age, but no significant reduction between five and six years [16]. Foot structure and function differ between younger and older adults [17]. Focusing on young adults may therefore limit potential variation associated with growth and ageing. Examining this age group may clarify how relative midfoot plantar contact relates to static foot posture, change in navicular height and rearfoot alignment. Analysis of individual FPI-6 components may also identify the specific features of foot posture associated with CSI. These findings may provide a basis for future comparative and longitudinal studies.

Relative midfoot plantar contact may also be associated with structural foot deformities. A study on older adults examined static foot posture and plantar pressure progression during walking. In the assessment of static foot posture, a low arch was associated with hammer and overlapping toes, whereas a pronated plantar pressure pattern during walking was associated with hallux valgus and overlapping toes [3]. Following adjustment for age, sex, height and weight, the latter association remained significant for overlapping toes but not for hallux valgus [3]. A moderate correlation between a radiographic measure of hallux valgus severity and a measure of flatfoot alignment has also been reported [18]. However, evidence regarding the associations of CSI with clinically assessed hallux valgus and lesser toe deformities in young adults remains limited.

The primary aim of this study was to examine the associations of CSI with FPI-6 total score, NDT and RCSP in young adults. Secondary aims were to examine the association between FPI-6 total score and NDT; evaluate the associations of CSI with individual FPI-6 components; assess agreement between CSI and FPI-6 classifications; and evaluate measurement reliability. Additional analyses examined the associations of CSI with clinically assessed hallux valgus and lesser toe deformities.

## 2. Materials and Methods

### 2.1. Study Design

A cross-sectional observational study was conducted to examine associations between footprint-derived medial longitudinal arch morphology, static foot posture, and clinically assessed foot deformities in young adults. Both feet of each participant were included as separate observations. Statistical analyses accounted for the correlation between the right and left feet of the same participant [19,20]. The study was reported in accordance with the Strengthening the Reporting of Observational Studies in Epidemiology (STROBE) guidelines for cross-sectional studies [21].

### 2.2. Ethical Approval

Ethical approval was obtained from the Institutional Review Board of the University of Jordan (Approval No. 20259371). All participants received information about the study procedures and provided written informed consent before participation. Data were coded and handled confidentially to protect participants’ privacy.

### 2.3. Study Setting and Participants

Participant recruitment and data collection were conducted between November 2025 and August 2026. A convenience sample of participants was recruited from the University of Jordan community through posters and recruitment notices displayed on university notice boards. The sample comprised all eligible individuals who consented to participate during the predefined recruitment period. No a priori sample size calculation was performed. All assessments were conducted at the clinic of the Department of Prosthetics and Orthotics, School of Rehabilitation Sciences, University of Jordan, Amman, Jordan.

Participants were considered eligible if they were aged 18 years or older, could stand and walk independently and were able to complete the required clinical and footprint assessments. Exclusion criteria were lower limb amputation, previous lower limb surgery, lower limb trauma with ongoing effects, or any musculoskeletal, neurological or systemic condition that substantially affected foot structure, posture, gait or weight bearing. All eligible participants who completed the required assessments during the recruitment period were included in the study.

### 2.4. Data Collection Procedure

**Figure 1:**
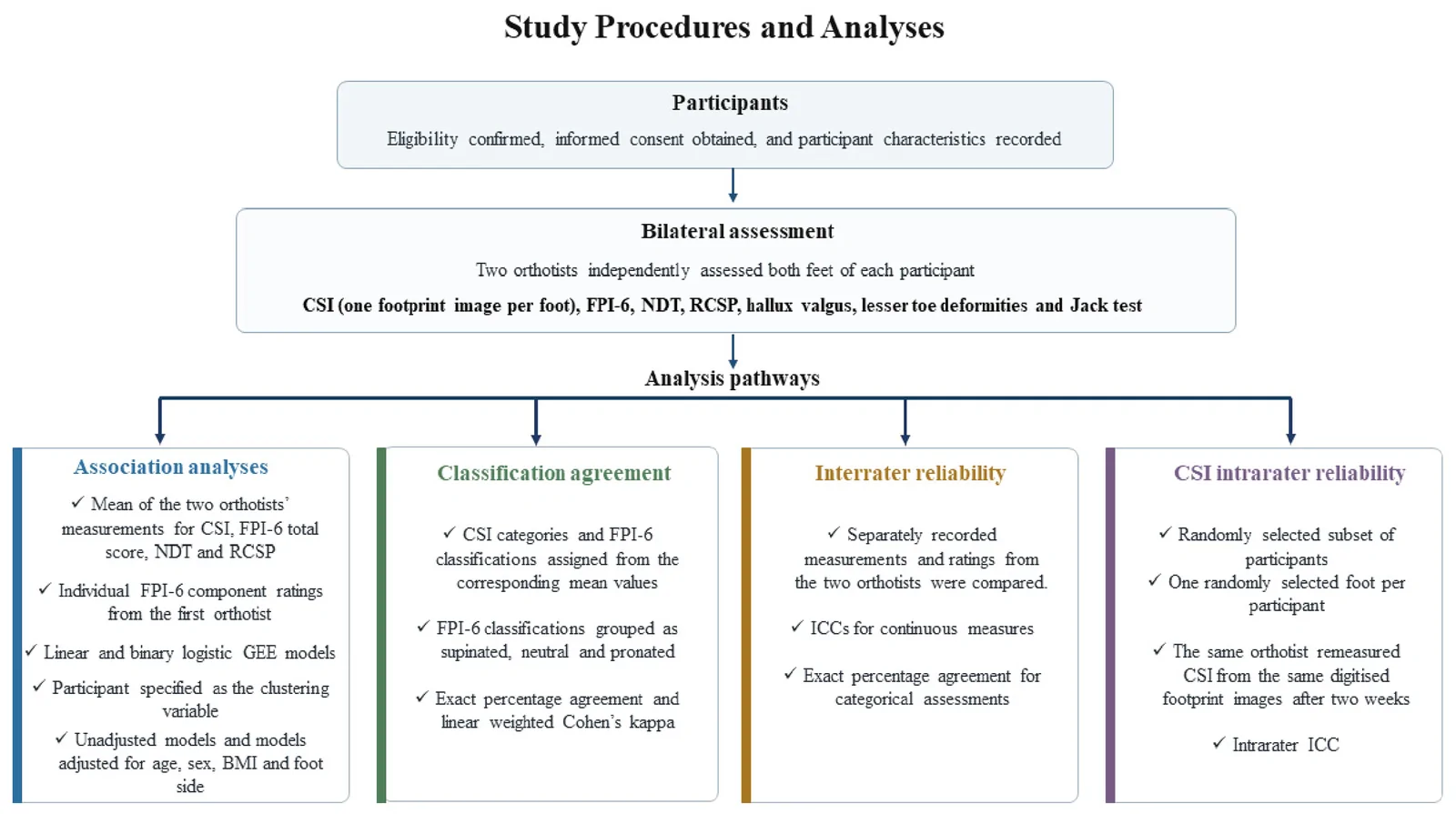
Overview of participant assessment and analysis pathways.

Participant characteristics, including age, sex, height, body mass and body mass index (BMI), were recorded before the foot assessments. Participants underwent a standardised barefoot assessment of both feet. An overview of participant assessment and analysis pathways is presented in Figure 1.

Each trained orthotist independently assessed both feet and recorded the findings without access to the other orthotist’s measurements or ratings. The assessment included FPI-6, CSI, NDT, RCSP, hallux valgus, lesser toe deformities and the Jack test. Measurements and clinical findings were recorded separately for the right and left feet.

The Jack test, also known as the Hubscher manoeuvre, was performed on all feet to observe the response of the medial longitudinal arch to hallux dorsiflexion. With the participant standing barefoot and distributing body weight evenly between both feet, the examiner passively dorsiflexed the hallux at the first metatarsophalangeal joint and observed the response of the medial longitudinal arch. Among feet classified as having a low arch footprint pattern, elevation of the arch indicated a flexible low arch, whereas absence of arch elevation indicated a rigid low arch [22].

#### 2.4.1. FPI-6

Static foot posture was assessed using the FPI-6. The instrument comprises six criterion-based observations: talar head palpation, curvature above and below the lateral malleolus, calcaneal position in the frontal plane, talonavicular joint prominence, medial longitudinal arch congruence, and forefoot position relative to the rearfoot. Each component is scored from −2, indicating a feature associated with supination, to +2, indicating a feature associated with pronation. The six component scores are summed to produce a total score ranging from −12 to +12, with higher scores indicating a more pronated foot posture [8,9].

The mean of the two orthotists’ FPI-6 total scores was used in the descriptive and primary association analyses to reduce measurement variation between orthotists. The individual component ratings were not averaged because this could produce scores that are not among the five permitted component values. Therefore, the component ratings recorded by the first orthotist were used in the secondary analyses of individual FPI-6 components.

The conventional FPI-6 classifications are defined as highly supinated (−12 to −5), supinated (−4 to −1), neutral (0 to +5), pronated (+6 to +9), and highly pronated (+10 to +12) [23]. Because the mean of the two orthotists’ total scores could produce half-point values, midpoint boundaries between the conventional categories were applied: highly supinated (<−4.5), supinated (−4.5 to <−0.5), neutral (−0.5 to <5.5), pronated (5.5 to <9.5), and highly pronated (≥9.5).

#### 2.4.2. CSI

Medial longitudinal arch morphology was characterised using CSI calculated from static ink footprints [24]. Participants stood barefoot with their body weight distributed evenly between both feet, and one footprint was obtained from each foot. Each footprint was inspected for quality before digitisation. A footprint was accepted when the boundaries required for measurement were clearly identifiable and there was no substantial smudging, movement artefact or double impression. If these criteria were not met, the footprint was repeated.

Each footprint was digitised at 300 dpi using a Canon flatbed scanner (Canon Inc., Tokyo, Japan) and saved as a PNG image without altering its original proportions. Digital measurements were performed using Fiji/ImageJ2, version 2.16.0 (ImageJ 1.54p), an open-source platform for scientific image analysis [25]. Both orthotists independently measured CSI from the same digitised footprint image for each foot and were blinded to each other’s measurements and the corresponding FPI-6, NDT and RCSP results.

**Figure 2:**
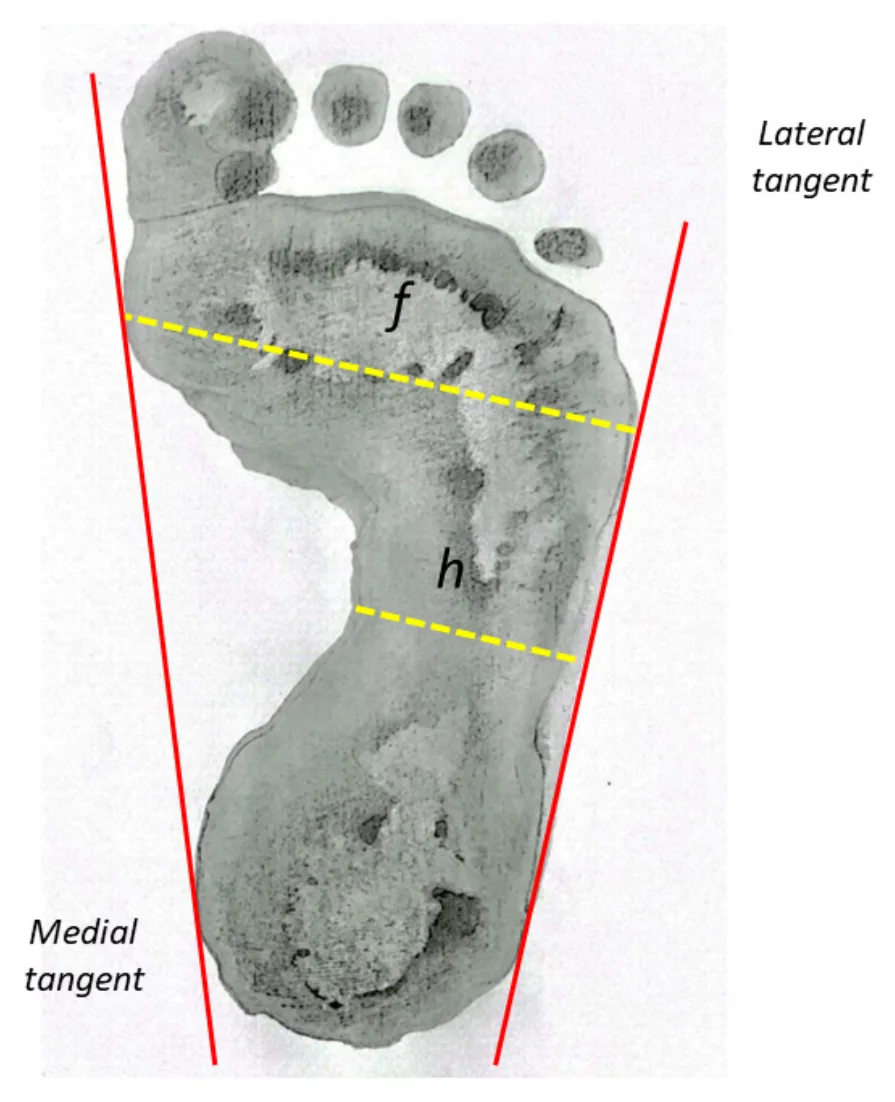
Measurement of the CSI from a representative static footprint. The medial and lateral tangents are shown in red. The maximum forefoot contact width (*f*) and minimum midfoot contact width (*h*) are shown in yellow.

On each digitised footprint image, a medial tangent was drawn between the most medial points of the heel and forefoot, and a lateral tangent was drawn between the most lateral points of the heel and forefoot. The maximum forefoot contact width (f) was measured across the widest continuous metatarsal contact region, excluding the toe impressions. The minimum midfoot contact width (h) was measured across the narrowest continuous region of the midfoot [6,7]. Both widths were measured along parallel lines perpendicular to the lateral tangent [16]. Measurement endpoints were positioned at the continuous footprint boundaries, excluding isolated ink marks and external smudging (Figure 2).

The CSI was calculated as:

$$\mathrm{CSI}\left(\%\right)=\frac{h}{f}\times 100$$

where *h* represents the minimum midfoot contact width and *f* represents the maximum forefoot contact width. Higher CSI values indicated greater relative midfoot contact and a lower footprint-derived medial longitudinal arch.

Published CSI classification thresholds vary across studies and populations [6,26]. In the present study, CSI values were classified as high arch (<25%), normal arch (25–45%) or low arch (>45%) footprint patterns. These boundaries were adapted from the published thresholds of ≤24%, 25–45% and ≥46%, respectively [6], to allow continuous percentage values to be classified without gaps. The published classification was selected as the basis for this adaptation because it had been evaluated against the talar–first metatarsal angle, a radiographic measure of medial longitudinal arch alignment [6]. These categories were used for the descriptive and agreement analyses and were not interpreted as diagnostic classifications. In the primary association analyses, CSI was analysed as a continuous variable.

Published guidance recommends at least 30 subjects for ICC estimation [27]. For the intrarater reliability assessment, 62 participants were randomly selected from the full sample. One foot was randomly selected from each participant, providing 62 feet for the analysis. After a two-week interval, the same orthotist repeated the CSI measurements using the same digitised footprint images without access to the initial measurements or calculated CSI values.

#### 2.4.3. NDT

Change in navicular height was assessed using the NDT described by Brody [10]. The navicular tuberosity was identified by palpation and marked. With the participant standing barefoot, the subtalar joint was positioned in neutral, and the vertical distance from the navicular tuberosity to the floor was measured in millimetres using a rigid ruler. The participant was then instructed to relax the feet while maintaining an upright standing position and distributing body weight evenly between both feet, and navicular height was measured again. Navicular drop was calculated by subtracting the height measured during relaxed standing from that measured while standing with the subtalar joint in neutral. Higher positive values indicated a greater reduction in navicular height. NDT was analysed as a continuous variable without applying a classification threshold because measurements depend on testing procedures and published interpretation criteria vary across studies [28,29].

#### 2.4.4. Resting Calcaneal Stance Position

Rearfoot alignment was assessed using RCSP [11]. With the participant lying prone and the feet extending beyond the end of the examination table, the medial and lateral borders of the posterior surface of the calcaneus were palpated at three levels. At each level, a point was marked midway between the medial and lateral borders. The three points were connected to establish the longitudinal bisection line of the calcaneus.

The participant then stood barefoot in a relaxed upright position, with body weight distributed evenly between both feet. A two-arm goniometer was positioned with one arm aligned with the posterior calcaneal bisection line and the other aligned with a vertical reference line perpendicular to the floor. The deviation from vertical was recorded in degrees. Calcaneal eversion, indicating valgus alignment, was recorded as a positive value; calcaneal inversion, indicating varus alignment, was recorded as a negative value; and vertical alignment was recorded as 0°.

#### 2.4.5. Hallux Valgus Assessment

The presence and severity of hallux valgus were assessed using the Manchester Scale developed by Garrow et al. [30]. This nonradiographic photographic scale comprises four reference photographs showing increasing levels of hallux valgus severity and has demonstrated interobserver reliability and validity against radiographic measurements [30,31]. With the participant standing barefoot in a relaxed position, each foot was visually compared with the photographs and classified as grade 1 (no hallux valgus), grade 2 (mild hallux valgus), grade 3 (moderate hallux valgus) or grade 4 (severe hallux valgus) [30].

For descriptive and association analyses, grade 1 was classified as absence of hallux valgus, whereas grades 2–4 were classified as presence of hallux valgus.

#### 2.4.6. Assessment of Lesser Toe Deformities

The second to fifth toes of each foot were examined for visible deformities while the participant stood barefoot in a relaxed position. Deformities were classified according to their clinical appearance. Hammer toe was defined as flexion of the proximal interphalangeal joint, with the distal interphalangeal joint in a neutral or extended position. Claw toe was defined as extension of the metatarsophalangeal joint with flexion of both the proximal and distal interphalangeal joints. Mallet toe was defined as isolated flexion of the distal interphalangeal joint [32]. Any other visible deformity of the lesser toes was recorded descriptively.

The presence or absence of hammer, claw and mallet toe was recorded separately for each toe. For the descriptive and association analyses, a composite variable was created for each foot. The variable was coded as present when hammer, claw or mallet toe was identified in at least one of the second to fifth toes and as absent when none of these deformities was identified.

### 2.5. Statistical Analysis

Statistical analyses were performed using IBM SPSS Statistics, version 25.0 (IBM Corp., Armonk, NY, USA). Participant-level bootstrap analyses were performed using Python, version 3.11 (Python Software Foundation, Wilmington, DE, USA). Continuous variables were summarised using means and standard deviations or medians and interquartile ranges, as appropriate. Categorical variables were summarised using frequencies and percentages. Statistical significance was set at *p* < 0.05.

Both feet were included as separate observations. Because measurements from the right and left feet of the same participant may be correlated, generalised estimating equation (GEE) models were used, with participants treated as clusters [19,20]. An exchangeable working correlation structure and robust standard errors were applied.

For the descriptive and association analyses, CSI, FPI-6 total score, NDT and RCSP were calculated as the mean of the two orthotists’ measurements. The mean CSI values and mean FPI-6 total scores were used to assign CSI categories and FPI-6 classifications, respectively. Individual FPI-6 component analyses used the ratings recorded by the first orthotist.

Separate linear GEE models were used to examine the associations of CSI with FPI-6 total score, NDT and RCSP. An additional linear GEE model was used to examine the association between FPI-6 total score and NDT. The linear models used a normal distribution. CSI was analysed as a continuous variable, and its coefficients were reported for each increase of 10 percentage points in CSI. Each association was examined before and after adjustment for age, sex, BMI and foot side. Additional models included interactions between CSI and sex to examine whether the associations of CSI with FPI-6 total score and NDT differed between male and female participants.

Binary logistic GEE models were used to examine the associations of CSI with hallux valgus, lesser toe deformities and individual FPI-6 component findings. For each FPI-6 component, scores indicating pronation (+1 or +2) were compared with scores of 0 or lower. Scores indicating supination (−1 or −2) were compared with scores of 0 or higher. Each component outcome was analysed separately using all feet. Associations were examined before and after adjustment for age, sex, BMI and foot side. Odds ratios (ORs) and robust 95% confidence intervals (CIs) were reported. The Benjamini–Hochberg procedure [33] was applied across the 12 component analyses to control the false discovery rate.

To correspond with the three CSI footprint pattern categories, the five FPI-6 classifications were combined into three ordered groups: supinated, comprising highly supinated and supinated; neutral; and pronated, comprising pronated and highly pronated. Agreement was assessed using exact percentage agreement and linear weighted Cohen’s kappa. Spearman’s rank correlation coefficients were used separately to describe the pairwise relationships among continuous CSI, FPI-6 total score and NDT.

Interrater reliability of CSI, FPI-6 total score, NDT and RCSP was assessed using two-way random effects, absolute agreement ICCs [27]. ICC(2,1) represented the reliability of a single orthotist’s measurement, whereas ICC(2,2) represented the reliability of the mean of the two orthotists’ measurements. Two-week intrarater reliability of CSI was assessed using a two-way mixed effects, absolute agreement, single-measure ICC, reported as ICC(A,1) [27]. The categorical ratings independently recorded by the two orthotists for Manchester Scale grade, Jack test classification and the presence or absence of claw, hammer and mallet toe were compared using exact percentage agreement. When the two orthotists assigned the same rating, that rating was used in the descriptive and association analyses. Any disagreement was planned to be resolved through discussion and consensus. To account for dependence between feet from the same participant, 95% CIs for the interrater ICCs and linear weighted Cohen’s kappa were estimated using 2000 participant-level bootstrap resamples. Both feet were retained for each selected participant.

## 3. Results

### 3.1. Participant Characteristics

The study included 350 participants, with measurements obtained from 700 feet. No data were missing for the variables included in the analyses. The sample comprised 191 females (54.6%) and 159 males (45.4%). Participant characteristics are summarised in Table 1.

### 3.2. Foot Morphology, Posture, and Deformities

Table 2 presents the descriptive results for the 700 feet assessed. Across all feet, the mean CSI was 42.54 ± 9.95%. Based on the selected CSI thresholds, 18 feet (2.6%) demonstrated a high arch footprint pattern, 413 (59.0%) a normal arch footprint pattern and 269 (38.4%) a low arch footprint pattern. A low arch footprint pattern was identified in 149 participants; 29 participants had this pattern in one foot and 120 had it in both feet. All 269 feet with a low arch footprint pattern demonstrated arch elevation during the Jack test and were therefore classified as flexible.

Across the 700 feet assessed, the mean FPI-6 total score was 4.35 ± 2.61. Eight feet (1.1%) were classified as highly supinated, eight (1.1%) as supinated, 445 (63.6%) as neutral, 236 (33.7%) as pronated and three (0.4%) as highly pronated. Overall, 239 feet from 146 participants were classified as pronated or highly pronated. This classification was present in one foot of 53 participants and in both feet of 93 participants.

The mean navicular drop was 12.20 ± 4.39 mm, and the mean RCSP was 5.04 ± 4.35°. Hallux valgus was identified in 26 feet from 23 participants, and all affected feet were classified as Manchester grade 2. A lesser toe deformity was identified in 14 feet from 10 participants: nine feet had claw toe, three had hammer toe and two had mallet toe. No other visible lesser toe deformities were observed.

Mean NDT increased across the CSI categories from high arch to low arch, while mean RCSP changed from varus alignment in the high arch category to progressively greater valgus alignment in the normal and low arch categories. Similarly, mean NDT and RCSP generally increased across the FPI-6 classifications from highly supinated to highly pronated. Descriptive values for NDT and RCSP according to CSI footprint pattern category and FPI-6 classification are presented in Table 3.

### 3.3. Measurement Reliability

Interrater reliability of a single measurement was good for FPI-6 total score, RCSP and CSI, with ICC(2,1) values ranging from 0.874 to 0.884. NDT demonstrated moderate reliability for a single measurement (ICC(2,1) = 0.644, 95% CI 0.618–0.666).

Reliability based on the mean of the two orthotists’ measurements was excellent for FPI-6 total score, RCSP and CSI, with ICC(2,2) values ranging from 0.933 to 0.938. Reliability of the mean NDT measurement was good (ICC(2,2) = 0.783, 95% CI 0.764–0.800) (Table 4).

Intrarater reliability of CSI over a two-week interval was excellent in 62 feet from 62 participants (ICC(A,1) = 0.989, 95% CI 0.977–0.994). Complete interrater agreement was observed for Manchester Scale grade, Jack test classification and ratings of the presence or absence of claw, hammer and mallet toe. The two orthotists assigned identical ratings across all 700 feet, corresponding to 100% agreement. Therefore, one rating per foot was used in the descriptive and association analyses.

### 3.4. Associations Between CSI and Clinical Measures

**Figure 3:**
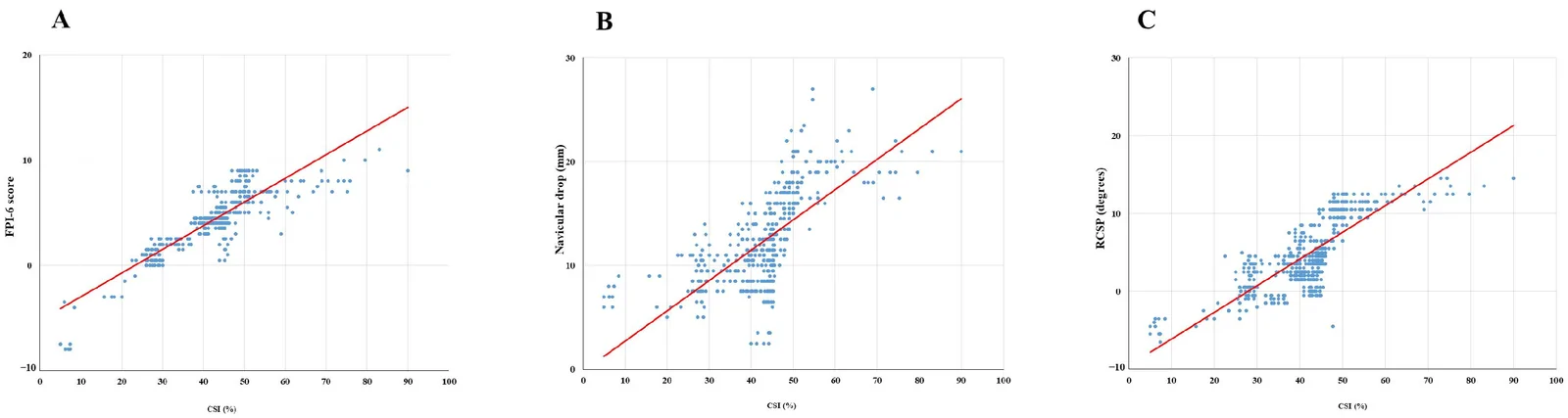
Associations between CSI and continuous clinical measures. Scatterplots show the relationships of CSI with (**A**) FPI-6 total score, (**B**) NDT and (**C**) RCSP. Each point represents one foot (*n* = 700). Red lines represent unadjusted linear trends and are included for descriptive visualisation. Adjusted GEE estimates accounting for the clustering of feet within participants and adjustment for age, sex, BMI and foot side are presented in Table 5.

In the adjusted linear GEE models, each 10-percentage-point increase in CSI was associated with a 2.141-point-higher FPI-6 total score (95% CI 1.901–2.382; *p* < 0.001), a 2.660 mm greater navicular drop (95% CI 2.309–3.011; *p* < 0.001) and an RCSP that was 3.240° more valgus (95% CI 2.953–3.527; *p* < 0.001). In a separate adjusted linear GEE model, each point increase in FPI-6 total score was associated with a 0.914 mm greater navicular drop (95% CI 0.766–1.063; *p* < 0.001). All models were adjusted for age, sex, BMI and foot side (Table 5; Figure 3). Additional analyses found no evidence that sex modified the associations of CSI with FPI-6 total score or NDT (interaction coefficients: *B* = −0.051, 95% CI −0.531 to 0.429, *p* = 0.836; and *B* = −0.003, 95% CI −0.684 to 0.677, *p* = 0.992, respectively).

Spearman’s rank correlation coefficients were positive between CSI and FPI-6 total score (ρ = 0.828), CSI and NDT (ρ = 0.726), and FPI-6 total score and NDT (ρ = 0.654). The corresponding unadjusted GEE estimates are provided in Supplementary Table S1.

### 3.5. Associations Between CSI, Hallux Valgus, and Lesser Toe Deformities

Adjusted logistic GEE analyses showed that each increase of 10 percentage points in CSI was associated with higher odds of hallux valgus (OR = 1.628, 95% CI 1.025–2.587; *p* = 0.039) and any lesser toe deformity (OR = 1.346, 95% CI 1.063–1.704; *p* = 0.014) (Table 6). The corresponding unadjusted estimates are provided in Supplementary Table S2.

Hallux valgus was identified in 26 feet from 23 participants, and all affected feet were classified as Manchester grade 2. At least one lesser toe deformity was identified in 14 feet from 10 participants. Because each deformity occurred in very few participants, only the association between CSI and the presence of any lesser toe deformity was analysed; the three deformities were not analysed separately.

### 3.6. Associations Between CSI and Individual FPI-6 Component Findings

Secondary analyses examined the associations between CSI and individual FPI-6 component findings. Each increase of 10 percentage points in CSI was positively associated with the talar head being palpable on the medial side (OR = 10.434, 95% CI 7.141–15.246), curvature above and below the lateral malleolus consistent with pronation (OR = 12.306, 95% CI 7.761–19.513), increased talonavicular joint prominence (OR = 5.331, 95% CI 3.819–7.440) and a lowered medial longitudinal arch (OR = 6.221, 95% CI 4.762–8.128).

Higher CSI was also positively associated with calcaneal valgus (OR = 2.199, 95% CI 1.784–2.709) and forefoot abduction (OR = 3.098, 95% CI 2.279–4.211). In contrast, higher CSI values were negatively associated with all six findings consistent with a more supinated foot posture. The adjusted ORs ranged from 0.012 for the talar head being palpable on the lateral side to 0.306 for calcaneal varus. Complete adjusted results are presented in Table 7, and the corresponding unadjusted estimates are provided in Supplementary Table S3.

All associations remained statistically significant after the Benjamini–Hochberg correction (all adjusted *p* < 0.001). Some findings, particularly reduced talonavicular joint prominence and the talar head being palpable on the lateral side, were observed in relatively few feet; therefore, their ORs should be interpreted cautiously.

### 3.7. Agreement Between CSI and FPI-6 Classifications

For the agreement analysis, the five FPI-6 classifications were combined into three ordered groups corresponding to the three CSI footprint pattern categories: supinated (highly supinated and supinated), neutral, and pronated (pronated and highly pronated). The original five FPI-6 classifications are reported separately in Table 2.

**Figure 4:**
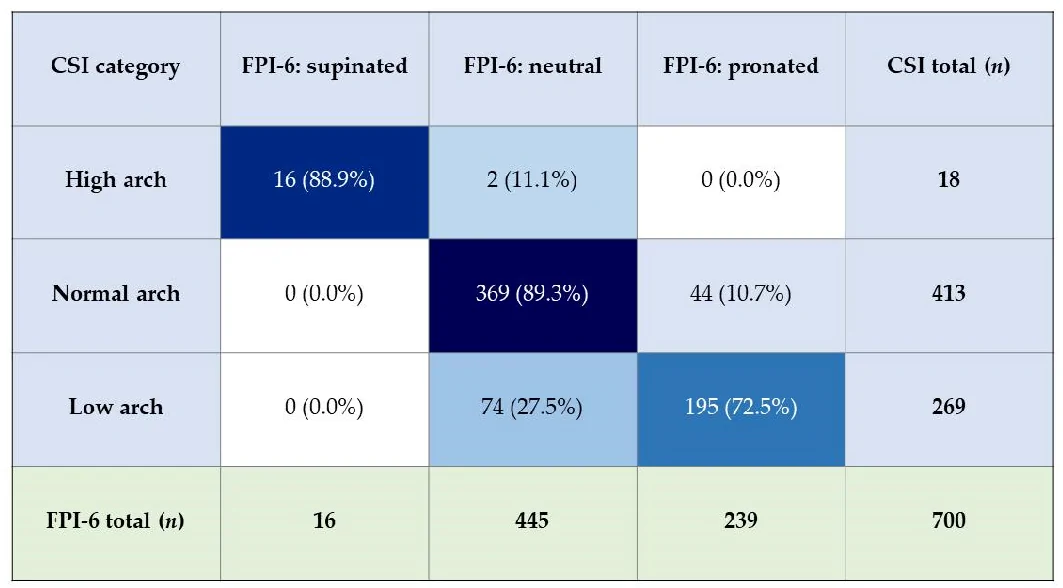
Distribution of grouped FPI-6 classifications within each CSI footprint pattern category. Values are presented as *n* (row percentage), with percentages calculated within each CSI category. Darker blue shading indicates a higher row percentage. Totals were not included in the colour scale.

The CSI footprint pattern categories and grouped FPI-6 classifications agreed for 580 of 700 feet (82.9%). Linear weighted Cohen’s kappa was 0.664 (95% participant-level bootstrap CI 0.595–0.727), indicating that agreement exceeded that expected by chance but remained incomplete. Among the 120 feet classified differently by the two methods, 74 were classified as having a low arch by CSI but as neutral by FPI-6, 44 were classified as having a normal arch by CSI but as pronated by FPI-6, and two were classified as having a high arch by CSI but as neutral by FPI-6 (Figure 4).

## 4. Discussion

This cross-sectional study examined the associations between relative midfoot plantar contact measured using CSI and clinical measures of foot posture in young adults. Higher CSI values were associated with a higher FPI-6 total score, greater navicular drop and a more valgus RCSP. A higher FPI-6 total score was also associated with greater navicular drop. The component analyses supported these findings, with higher CSI positively associated with FPI-6 features indicating a more pronated foot posture and negatively associated with features indicating a more supinated posture. CSI footprint pattern categories and grouped FPI-6 classifications agreed for most feet, although agreement remained incomplete. The associations of CSI with hallux valgus and lesser toe deformities should be interpreted cautiously because few participants had these conditions. Interrater reliability was good for FPI-6 total score, CSI and RCSP and moderate for NDT, while CSI demonstrated excellent intrarater reliability over two weeks.

### 4.1. Associations of CSI with FPI-6, NDT and RCSP

The mean CSI was 42.54%. Based on the selected CSI thresholds, 38.4% of feet were classified as having a low arch footprint pattern. According to the FPI-6 classifications, 34.1% of feet were classified as pronated or highly pronated. These classifications represent different characteristics of the foot and should not be considered equivalent. The proportion classified as having a low arch also depends on the CSI thresholds applied. CSI quantifies relative midfoot plantar contact, whereas FPI-6 evaluates static foot posture through observations of the rearfoot, midfoot and forefoot.

The mean FPI-6 total score was 4.35, similar to the mean score previously reported in healthy adults [9]. Although this value was within the neutral FPI-6 category, it was towards the upper boundary of that category and close to the threshold for a pronated foot posture.

Higher CSI values were associated with higher FPI-6 total scores. After adjustment for age, sex, BMI and foot side, an increase of 10 percentage points in CSI was associated with an estimated increase of 2.141 points in FPI-6 total score. This finding is consistent with the characteristics assessed by the two measures: greater CSI values reflect greater relative midfoot plantar contact, whereas higher FPI-6 total scores indicate a greater number or magnitude of clinical features associated with a pronated foot posture.

The positive correlation between CSI and FPI-6 total score is consistent with findings reported in young adults with low arch feet [12] and among children aged 5–8 years with different arch morphologies [13]. The correlation observed in the present study was stronger than that previously reported in young adults with low arch feet [12]. This difference may partly reflect the inclusion of participants with a broader range of foot postures in the present study, whereas the previous study was restricted to young adults with a low arch [12]. Direct comparisons should therefore be made cautiously.

Higher CSI values were associated with greater navicular drop. After adjustment for age, sex, BMI and foot side, each increase of 10 percentage points in CSI was associated with an estimated 2.660 mm greater navicular drop (95% CI 2.309–3.011). Similarly, each point increase in FPI-6 total score was associated with an estimated 0.914 mm greater navicular drop (95% CI 0.766–1.063). These findings are consistent with previously reported positive relationships between CSI and NDT [7,12] and between FPI-6 and NDT [12,13]. Overall, the findings suggest that greater relative midfoot plantar contact and a more pronated static foot posture may be related to a greater change in navicular height between subtalar neutral and relaxed standing.

There was no evidence that sex modified the associations of CSI with FPI-6 total score or NDT in the present study. Previous research reported no significant sex difference in FPI-6 scores [9] or navicular drop among adults aged 18–21 years [34]. A recent study reported no significant sex differences in CSI, the Staheli Index or the Arch Index, whereas Clarke’s angle was higher in females [35]. These studies compared measurement values between males and females rather than examining whether sex modified the associations between measures. Therefore, the present findings do not establish that the associations are identical between sexes, but indicate that differences related to sex were not detected within this sample.

RCSP also varied across the CSI footprint pattern categories. Mean RCSP was 3.47° of varus among high arch feet, 2.71° of valgus among normal arch feet and 9.20° of valgus among low arch feet. Consistent with these descriptive findings, each increase of 10 percentage points in CSI was associated with an estimated RCSP that was 3.240° more valgus after adjustment for age, sex, BMI and foot side (95% CI 2.953–3.527). In contrast, a previous study found no significant correlations between CSI and RCSP within either normal arch or flatfoot groups defined using NDT [36]. The different findings may partly reflect differences in how calcaneal alignment was measured and foot arch type was classified. The present findings suggest that greater relative midfoot plantar contact may be related to a more valgus calcaneal position during relaxed standing.

### 4.2. Associations of CSI with Individual FPI-6 Components

The component analyses provided further information about the relationship between CSI and static foot posture. The individual FPI-6 components represent clinical features associated with supinated or pronated foot posture [8]. In the present study, higher CSI values were associated with the talar head being palpable on the medial side, curvature above and below the lateral malleolus consistent with pronation, calcaneal valgus, increased talonavicular joint prominence, a lowered medial longitudinal arch and forefoot abduction. Conversely, higher CSI values were associated with a lower likelihood of the talar head being palpable on the lateral side, curvature above and below the lateral malleolus consistent with supination, calcaneal varus, reduced talonavicular joint prominence, a higher medial longitudinal arch and forefoot adduction.

The largest estimates were observed for the talar head being palpable on the medial side and for curvature above and below the lateral malleolus consistent with pronation. These findings may suggest that relative midfoot plantar contact is related to several clinical features assessed by FPI-6. Associations were observed across rearfoot, midfoot and forefoot components, although their magnitude varied among the individual findings.

Associations for all FPI-6 components remained statistically significant after correction for multiple testing. Nevertheless, these analyses were secondary, and some component findings were observed in a relatively small number of feet. Estimates based on these findings should therefore be interpreted cautiously.

### 4.3. Agreement Between CSI and FPI-6 Classifications

The CSI footprint pattern categories and grouped FPI-6 classifications agreed for 580 of 700 feet (82.9%). Linear weighted Cohen’s kappa was 0.664 (95% participant-level bootstrap CI 0.595–0.727), suggesting that agreement exceeded that expected by chance but remained incomplete. Among low arch feet, 72.5% were classified as pronated and 27.5% as neutral by FPI-6. The most frequent classification difference involved feet classified as low arch by CSI but neutral by FPI-6, followed by feet classified as normal arch by CSI but pronated by FPI-6.

The incomplete agreement may reflect differences in the characteristics assessed by the two methods. CSI is derived from a two-dimensional static footprint and quantifies relative midfoot plantar contact, whereas FPI-6 evaluates static foot posture through clinical observations of the rearfoot, midfoot and forefoot. A foot may therefore demonstrate greater midfoot plantar contact without showing sufficient clinical features to be classified as pronated by FPI-6. Conversely, a foot may demonstrate clinical features associated with pronation without meeting the CSI threshold for a low arch footprint pattern. Previous research has similarly shown that static foot assessment methods may classify the same foot differently [5]. However, direct comparison is limited because that study compared FPI-6 with NDT and clinical angular measurements rather than CSI [5].

Categorising continuous measurements may place feet with similar values into different groups when their measurements fall on opposite sides of a classification threshold. In addition, published footprint classification methods and thresholds vary across assessment methods and populations [4,26]. Therefore, the percentage agreement and weighted kappa observed in the present study are specific to the classification scheme applied. Despite the incomplete categorical agreement, CSI and FPI-6 total scores were positively correlated (ρ = 0.828), suggesting that the two continuous measures generally changed in the same direction. Correlation describes the relationship between continuous scores, whereas weighted kappa describes the extent to which individual feet are assigned to corresponding ordered categories. These findings should therefore be considered complementary and interpreted according to the specific question addressed by each analysis.

### 4.4. Hallux Valgus and Lesser Toe Deformities

Hallux valgus was identified in 26 feet (3.7%) from 23 participants, and all affected feet were classified as mild according to the Manchester Scale. When CSI was analysed as a continuous variable, an increase of 10 percentage points in CSI corresponded to an estimated 62.8% increase in the adjusted odds of hallux valgus (OR = 1.628, 95% CI 1.025–2.587; *p* = 0.039). This finding is broadly consistent with previous evidence reporting associations between low arch or flatfoot alignment and hallux valgus in selected adult populations [18,37]. However, hallux valgus is multifactorial and has been associated with demographic, hereditary and structural factors [38].

This association should be considered preliminary. Only 23 participants had hallux valgus, all affected feet were classified as mild, and no moderate or severe cases were observed. The confidence interval was relatively wide, with its lower limit close to the null value. Furthermore, CSI was analysed as a continuous measure; therefore, the finding represents an association between greater relative midfoot plantar contact and mild hallux valgus rather than an association specifically involving the low arch CSI category. The cross-sectional design also precludes conclusions about whether greater relative midfoot plantar contact contributed to the development of hallux valgus.

Lesser toe deformities were identified in 14 feet (2.0%) from 10 participants. An increase of 10 percentage points in CSI corresponded to an estimated 34.6% increase in the adjusted odds of having any lesser toe deformity (OR = 1.346, 95% CI 1.063–1.704; *p* = 0.014). However, the unadjusted association was not statistically significant. The small number of affected participants relative to the number of covariates, together with the difference between the unadjusted and adjusted findings, suggests that the estimate may be sensitive to the model specification. This association should therefore be considered exploratory and interpreted cautiously. Separate associations for claw, hammer and mallet toe were not examined because these deformities affected only nine, three and two feet, respectively.

The low frequencies of hallux valgus and lesser toe deformities may partly reflect the young age of the sample. Differences in foot structure, function and structural foot deformities have been reported between younger and older adults [17], while hallux valgus and toe deformities were common in older adult samples [39,40]. Consequently, the findings from this sample of young adults recruited from a university community should not be generalised to older adults or clinical populations with more frequent or severe foot deformities.

### 4.5. Measurement Reliability

Interrater reliability of a single measurement was good for FPI-6, CSI and RCSP and moderate for NDT. Reliability based on the mean of the two orthotists’ measurements was higher for all four measures, reaching excellent levels for FPI-6, CSI and RCSP and a good level for NDT. This finding is consistent with the generally higher ICCs obtained for averaged rather than single measurements [27]. These findings support the use of the mean of the two orthotists’ measurements in the primary analyses.

CSI also demonstrated excellent two-week intrarater reliability in the subset of 62 feet. Because the same footprint images were remeasured, this analysis assessed the consistency of digital landmark placement and CSI calculation rather than the repeatability of footprint acquisition. The clearly defined measurement landmarks and standardised digital procedure may have contributed to the high reliability.

Examiner training and the use of standardised assessment procedures may have contributed to the good reliability of FPI-6 and RCSP. Previous studies have reported different FPI-6 reliability estimates across participant groups and assessment settings [41,42]. In contrast, the lower single-measure reliability of NDT may reflect difficulties in consistently identifying the navicular tuberosity, positioning the subtalar joint in neutral and maintaining comparable standing posture and weight distribution. Variation in the reliability of navicular drop measurements has also been reported [7,28]. The moderate ICC(2,1) suggests that small differences in NDT based on an individual orthotist’s measurement should be interpreted cautiously. Reliability was higher when the mean of the two orthotists’ measurements was used.

Complete exact agreement was observed between the two orthotists for Manchester Scale grade, Jack test classification and ratings of claw, hammer and mallet toe. Although this finding indicates consistency between the orthotists, hallux valgus and lesser toe deformities were uncommon; therefore, agreement for these assessments was based mainly on feet without deformity. The findings provide limited information about agreement when these deformities are present. Furthermore, exact percentage agreement describes consistency between the orthotists but does not establish the validity of the categorical assessments.

### 4.6. Clinical and Research Implications

The findings of the present study suggest that footprint and clinical assessment methods describe related but different characteristics of the foot. CSI provides a quantitative description of relative midfoot plantar contact, FPI-6 evaluates static foot posture across the rearfoot, midfoot and forefoot, NDT measures change in navicular height, and RCSP describes frontal plane calcaneal alignment during relaxed standing. The association and incomplete classification agreement between CSI and FPI-6 suggest that these methods may provide complementary information, and the results obtained using one method may not necessarily correspond to those obtained using the other.

The selection of an assessment method may therefore be guided by the characteristic of interest. CSI may be considered when relative midfoot plantar contact is being evaluated, whereas FPI-6 may be used when a broader clinical assessment of static foot posture is required. NDT and RCSP may provide further information about change in navicular height and calcaneal alignment, respectively. Interpretation should remain specific to the characteristic assessed by each method, particularly when categorical thresholds are applied.

### 4.7. Limitations

Several limitations should be considered when interpreting the findings. The cross-sectional design does not permit conclusions about causality or the temporal relationships among relative midfoot plantar contact, static foot posture and structural foot deformities. Participants were young adults recruited from a university community, which may limit the generalisability of the findings to children, older adults, symptomatic individuals, athletes and people with rigid or more severe foot deformities.

CSI is a two-dimensional measure of plantar contact rather than a direct measure of skeletal alignment. Its value may be influenced by plantar soft tissue and weight distribution during footprint acquisition. Radiographic measurements were not available to evaluate skeletal arch alignment or quantify the hallux valgus angle. In addition, hallux valgus and lesser toe deformities were uncommon, resulting in estimates that may be imprecise or sensitive to the covariates included in the adjusted models.

The analyses of individual FPI-6 components involved multiple comparisons and were secondary, although the Benjamini–Hochberg procedure was applied to control the false discovery rate. Two-week intrarater reliability of CSI was assessed by remeasuring the same footprint images; therefore, it reflects the consistency of digital landmark placement and CSI calculation but does not assess the repeatability of footprint acquisition. Finally, the static assessments used in this study may not fully represent foot posture or function during gait and other dynamic activities.

Future studies involving broader age groups and clinical populations may help determine whether these relationships are consistent across populations. Longitudinal studies and comparisons with medical imaging or dynamic assessments may provide further information about the clinical interpretation of relative midfoot plantar contact and its relationship with foot posture and structural foot deformities.

## 5. Conclusions

In this sample of young adults, higher CSI values were associated with higher FPI-6 total scores, greater navicular drop and a more valgus RCSP. CSI and FPI-6 classifications showed general but incomplete agreement, suggesting that relative midfoot plantar contact and static foot posture provide related but different information. Associations of CSI with mild hallux valgus and lesser toe deformities should be interpreted cautiously because these conditions were uncommon.

Interrater reliability was good for individual measurements of FPI-6, CSI and RCSP and moderate for NDT, while reliability was higher when the mean of the two orthotists’ measurements was used. Digital remeasurement of CSI from the same footprint images also demonstrated excellent intrarater reliability. The findings support selecting and interpreting each assessment method according to the specific foot characteristic being evaluated.

## Figures and Tables

**Table 1:** Participant characteristics (*n* = 350 participants).

Characteristic	Mean ± SD	Median (IQR)	Range
Age (years)	20.75 ± 2.25	20.00 (19.00–22.00)	18.00–29.00
Body mass (kg)	63.36 ± 12.52	60.00 (55.00–73.00)	40.00–116.00
Height (m)	1.651 ± 0.075	1.64 (1.59–1.70)	1.46–1.86
BMI (kg/m^2^)	23.26 ± 4.38	22.50 (20.31–25.96)	12.54–42.61
Sex (*n* (%))			
Female	191 (54.6%)		
Male	159 (45.4%)		

**Table 2:** Foot measurements, classification categories, and deformities (*n* = 700 feet from 350 participants).

**Variable**	Value
Continuous foot measurements (mean ± SD (range))	
CSI (%)	42.54 ± 9.95 (5.00–90.02)
FPI-6 total score	4.35 ± 2.61 (−8.00–11.00)
NDT (mm)	12.20 ± 4.39 (2.50–27.00)
RCSP (degrees)	5.04 ± 4.35 (−6.50–14.50)
CSI footprint pattern category	
High arch	18 feet (2.6%); 11 participants (3.1%: 4 unilateral, 7 bilateral)
Normal arch	413 feet (59.0%); 222 participants (63.4%: 31 unilateral, 191 bilateral)
Low arch	269 feet (38.4%); 149 participants (42.6%: 29 unilateral, 120 bilateral)
FPI-6 classification	
Highly supinated	8 feet (1.1%); 4 participants (1.1%: 0 unilateral, 4 bilateral)
Supinated	8 feet (1.1%); 5 participants (1.4%: 2 unilateral, 3 bilateral)
Neutral	445 feet (63.6%); 248 participants (70.9%: 51 unilateral, 197 bilateral)
Pronated	236 feet (33.7%); 145 participants (41.4%: 54 unilateral, 91 bilateral)
Highly pronated	3 feet (0.4%); 2 participants (0.6%: 1 unilateral, 1 bilateral)
Hallux valgus (Manchester Scale)	
Grade 1: no hallux valgus	674 feet (96.3%)
Grade 2: mild hallux valgus	26 feet (3.7%); 23 participants (6.6%: 20 unilateral, 3 bilateral)
Grade 3: moderate hallux valgus	0 feet (0.0%); 0 participants (0.0%)
Grade 4: severe hallux valgus	0 feet (0.0%); 0 participants (0.0%)
Lesser toe deformities	
Claw toe	9 feet (1.3%); 5 participants (1.4%: 1 unilateral, 4 bilateral)
Hammer toe	3 feet (0.4%); 3 participants (0.9%: 3 unilateral, 0 bilateral)
Mallet toe	2 feet (0.3%); 2 participants (0.6%: 2 unilateral, 0 bilateral)
Any lesser toe deformity	14 feet (2.0%); 10 participants (2.9%)

**Table 3:** Descriptive NDT and RCSP values according to CSI footprint pattern category and FPI-6 classification.

Classification	Feet (*n*)	NDT (mm) Mean ± SD	RCSP (Degrees) Mean ± SD
**CSI footprint pattern category**			
High arch	18	7.56 ± 1.72	−3.47 ± 2.37
Normal arch	413	9.92 ± 2.68	2.71 ± 2.38
Low arch	269	16.02 ± 3.85	9.20 ± 3.11
**FPI-6 classification**			
Highly supinated	8	7.00 ± 0.76	−4.88 ± 1.06
Supinated	8	7.25 ± 1.67	−3.19 ± 0.96
Neutral	445	10.29 ± 2.93	2.93 ± 2.52
Pronated	236	16.05 ± 4.05	9.55 ± 2.98
Highly pronated	3	20.67 ± 1.53	12.83 ± 0.58

Values represent the mean of the two orthotists’ measurements. Positive RCSP values indicate valgus alignment, and negative values indicate varus alignment.

**Table 4:** Interrater reliability of continuous foot measurements (*n* = 700 feet).

Measure	ICC(2,1) (95% Bootstrap CI)	ICC(2,2) (95% Bootstrap CI)
FPI-6 score	0.874 (0.865–0.880)	0.933 (0.928–0.936)
NDT (mm)	0.644 (0.618–0.666)	0.783 (0.764–0.800)
RCSP (degrees)	0.878 (0.870–0.886)	0.935 (0.930–0.940)
CSI (%)	0.884 (0.878–0.890)	0.938 (0.935–0.942)

ICC: intraclass correlation coefficient; CI: confidence interval. ICC(2,1) represents the reliability of a single orthotist’s measurement; ICC(2,2) represents the reliability of the mean of the two orthotists’ measurements. Confidence intervals were estimated using participant-level bootstrap resampling, with both feet retained for each selected participant.

**Table 5:** Adjusted GEE associations among CSI, FPI-6 total score, NDT, and RCSP.

Predictor	Outcome	B	Robust SE	95% CI	*p* Value
CSI	FPI-6 total score	2.141	0.123	1.901–2.382	<0.001
CSI	NDT (mm)	2.660	0.179	2.309–3.011	<0.001
CSI	RCSP (degrees)	3.240	0.146	2.953–3.527	<0.001
FPI-6 total score	NDT (mm)	0.914	0.076	0.766–1.063	<0.001

B: unstandardised regression coefficient; SE: standard error. CSI coefficients represent the change in each outcome for every 10-percentage-point increase in CSI. The FPI-6 coefficient represents the change in NDT for each point increase in FPI-6 total score. Models were adjusted for age, sex, body mass index and foot side. Robust standard errors accounted for the correlation between the right and left feet of the same participant.

**Table 6:** Adjusted GEE associations between CSI, hallux valgus, and lesser toe deformities.

Outcome	Affected Feet/Participants (*n*/*n*)	Adjusted OR	95% CI	*p* Value
Mild hallux valgus * (Manchester grade 2)	26/23	1.628	1.025–2.587	0.039
Any lesser toe deformity	14/10	1.346	1.063–1.704	0.014

* Hallux valgus was defined analytically as Manchester grades 2–4 versus grade 1; however, all affected feet in this sample were grade 2. OR: odds ratio. ORs are reported for each increase of 10 percentage points in CSI. Models were adjusted for age, sex, body mass index and foot side.

**Table 7:** Adjusted GEE associations between CSI and individual FPI-6 component findings.

FPI-6 Component	Component Finding	Feet (*n*)	Adjusted OR	95% CI	*p* Value
Talar head palpation	Lateral palpation	28	0.012	0.003–0.042	<0.001
	Medial palpation	571	10.434	7.141–15.246	<0.001
Curvature above and below the lateral malleolus	Curvature indicating supination	33	0.024	0.004–0.135	<0.001
	Curvature indicating pronation	573	12.306	7.761–19.513	<0.001
Calcaneal position	Varus	84	0.306	0.240–0.389	<0.001
	Valgus	448	2.199	1.784–2.709	<0.001
Talonavicular joint prominence	Reduced prominence	13	0.035	0.012–0.107	<0.001
	Increased prominence	556	5.331	3.819–7.440	<0.001
Medial longitudinal arch congruence	Higher arch	46	0.278	0.189–0.410	<0.001
	Lowered arch	359	6.221	4.762–8.128	<0.001
Forefoot position	Adduction	30	0.225	0.152–0.333	<0.001
	Abduction	420	3.098	2.279–4.211	<0.001

Each FPI-6 component was analysed as two separate binary outcomes using all 700 feet. Scores above 0 were compared with scores of 0 or below, and scores below 0 were compared with scores of 0 or above. ORs are reported for each increase of 10 percentage points in CSI. Models accounted for the clustering of feet within participants and were adjusted for age, sex, BMI and foot side. The Benjamini–Hochberg procedure was applied across the 12 component analyses to control the false discovery rate.

## Data Availability

Data supporting the findings of this study are available from the corresponding authors upon reasonable request.

## Supplementary Materials

The following supporting information can be downloaded at: https://www.mdpi.com/article/10.3390/diagnostics16213455/s1, Table S1: Unadjusted GEE associations among CSI, FPI-6 total score, NDT, and RCSP; Table S2: Unadjusted GEE associations of CSI with hallux valgus and lesser toe deformities; Table S3: Unadjusted GEE associations of CSI with individual FPI-6 component findings.

## Abbreviations

The following abbreviations are used in this manuscript:

BMI	Body Mass Index
CI	Confidence Interval
CSI	Chippaux–Smirak Index
FPI-6	Six-item Foot Posture Index
GEE	Generalised Estimating Equation
ICC	Intraclass Correlation Coefficient
IQR	Interquartile Range
MLA	Medial Longitudinal Arch
NDT	Navicular Drop Test
OR	Odds Ratio
RCSP	Resting Calcaneal Stance Position
SD	Standard Deviation
SE	Standard Error
