# Supplementary material for: Associations Between the Chippaux–Smirak Index and Clinical Foot Assessments in Young Adults: A Cross-Sectional Study
Source: Diagnostics (Basel). 2026 Oct 22;16(21):3455. doi: 10.3390/diagnostics16213455 (PMC13761712; doi:10.3390/diagnostics16213455)
Supplement: Supplementary file 1 [file diagnostics-16-03455-s001.zip › diagnostics-4615167-supplementary.pdf]

Table S1. Unadjusted GEE associations among CSI, FPI-6 total score, NDT and RCSP.

Predictor	Outcome	Unadjusted B	Robust SE	95% CI	*p* value
**CSI**	**FPI-6 total score**	2.164	0.125	1.920–2.408	<0.001
**CSI**	**NDT (mm)**	2.726	0.174	2.384–3.068	<0.001
**CSI**	**RCSP (degrees)**	3.278	0.145	2.993–3.563	<0.001
**FPI-6 total score**	**NDT (mm)**	0.940	0.074	0.795–1.085	<0.001

B: unstandardised regression coefficient; SE: standard error; CI: confidence interval. CSI coefficients represent the change in the outcome per 10 percentage point increase in CSI. The FPI-6 coefficient represents the change in NDT per one point increase in FPI-6 total score. Estimates were obtained from unadjusted GEE models with participant specified as the clustering variable, an exchangeable working correlation structure and robust standard errors.

Table S2. Unadjusted GEE associations of CSI with hallux valgus and lesser toe deformities.

Outcome	Affected feet/participants (n/n)	Unadjusted OR	95% CI	***p* value**
Mild hallux valgus* (Manchester grade 2)	26/23	1.670	0.998–2.794	0.051
Any lesser toe deformity	14/10	1.128	0.789–1.614	0.509

OR: odds ratio; CI: confidence interval. ORs are reported per 10-percentage-point increase in CSI. Estimates were obtained from unadjusted generalised estimating equation models with participant specified as the clustering variable, an exchangeable working correlation structure and robust standard errors.
* Hallux valgus was defined analytically as Manchester grades 2–4 versus grade 1; however, all affected feet in this sample were grade 2.

Table S3. Unadjusted GEE associations of CSI with individual FPI-6 component findings.

FPI-6 component	Component finding	**Feet (*n*)**	Unadjusted OR	95% CI	*p* value
**Talar head palpation**	Lateral palpation	28	0.023	0.010–0.055	<0.001
	Medial palpation	571	10.011	6.993–14.332	<0.001
**Curvature above and below the lateral malleolus**	Curvature indicating supination	33	0.033	0.006–0.175	<0.001
	Curvature indicating pronation	573	12.159	7.724–19.139	<0.001
**Calcaneal position**	Varus	84	0.325	0.259–0.408	<0.001
	Valgus	448	2.195	1.796–2.684	<0.001
**Talonavicular joint prominence**	Reduced prominence	13	0.041	0.008–0.204	<0.001
	Increased prominence	556	5.413	3.914–7.486	<0.001
**Medial longitudinal arch congruence**	Higher arch	46	0.264	0.178–0.391	<0.001
	Lowered arch	359	6.275	4.832–8.149	<0.001
**Forefoot position**	Adduction	30	0.240	0.165–0.350	<0.001
	Abduction	420	3.116	2.301–4.221	<0.001

Each FPI-6 component was analysed as two separate binary outcomes using all 700 feet. Positive scores (+1 or +2) were compared with non-positive scores (−2, −1, or 0), and negative scores (−2 or −1) were compared with non-negative scores (0, +1, or +2). ORs are reported per 10 percentage point increase in CSI.
